# The dimerized pentraxin-like domain of the adhesion G protein–coupled receptor 112 (ADGRG4) suggests function in sensing mechanical forces

**DOI:** 10.1016/j.jbc.2023.105356

**Published:** 2023-10-18

**Authors:** Björn Kieslich, Renato H. Weiße, Jana Brendler, Albert Ricken, Torsten Schöneberg, Norbert Sträter

**Affiliations:** 1 Institute of Bioanalytical Chemistry, Center for Biotechnology and Biomedicine, Leipzig University, Leipzig, Germany; 2 Rudolf Schönheimer Institute of Biochemistry, Medical Faculty, Leipzig University, Leipzig, Germany; 3 Institute of Anatomy, Medical Faculty, Leipzig University, Leipzig, Germany

**Keywords:** adhesion G protein-coupled receptor, pentraxin, X-ray structure, signal transduction, homodimerization, small-angle X-ray scattering, X-ray crystallography, adhesion GPCR, GPR112, extracellular matrix, mucin, enterochromaffin cells, Paneth cells

## Abstract

Adhesion G protein–coupled receptors (aGPCRs) feature large extracellular regions with modular domains that often resemble protein classes of various function. The pentraxin (PTX) domain, which is predicted by sequence homology within the extracellular region of four different aGPCR members, is well known to form pentamers and other oligomers. Oligomerization of GPCRs is frequently reported and mainly driven by interactions of the seven-transmembrane region and N or C termini. While the functional importance of dimers is well-established for some class C GPCRs, relatively little is known about aGPCR multimerization. Here, we showcase the example of ADGRG4, an orphan aGPCR that possesses a PTX-like domain at its very N-terminal tip, followed by an extremely long stalk containing serine-threonine repeats. Using X-ray crystallography and biophysical methods, we determined the structure of this unusual PTX-like domain and provide experimental evidence for a homodimer equilibrium of this domain which is Ca^2+^-independent and driven by intermolecular contacts that differ vastly from the known soluble PTXs. The formation of this dimer seems to be conserved in mammalian ADGRG4 indicating functional relevance. Our data alongside of theoretical considerations lead to the hypothesis that ADGRG4 acts as an *in vivo* sensor for shear forces in enterochromaffin and Paneth cells of the small intestine.

Although it is generally accepted that G protein–coupled receptors (GPCRs) exist as monomers, there is convincing evidence that some GPCRs can also form dimers and even higher-order oligomers. Members of the metabotropic glutamate receptor-like GPCRs (class C) are well-known examples for forming stable homodimers and heterodimers (1). Moreover, transient oligomerization was frequently reported for rhodopsin-like (class A) receptors (2, 3). In class B GPCRs, the secretin receptor was one of the first members for which dimerization was described (4), yet other reports about homooligomerization and heterooligomerization followed (5). Within the class B, adhesion GPCRs (aGPCRs) form a cluster of structurally separate receptors comprising 33 members in the human genome, which can be further subdivided into nine families (6). Here, homodimerization and heterodimerization was shown in *trans* and *cis*, mediated *via* their large extracellular region (ECR) or the 7TM domain–containing C-terminal fragment. Disulfide bond–mediated homodimerization was observed for ADGRF5 (GPR116) (7). There is also some evidence for ECR-dependent homodimerization of the *Caenorhabditis elegans* LAT-1, which requires the presence of the N-terminal RBL domain (8). Moreover, the aGPCR members ADGRC1–3 undergo homophilic *trans* interactions (9, 10, 11). ADGRE5 heterodimerizes with lysophosphatidic acid receptor 1 in prostate and thyroid cancer cell lines and thus amplifies RhoA activation (12, 13). C-terminal fragment–mediated homodimerization was reported for ADGRL1 (latrophilin) upon latrotoxin treatment (14) as well as for ADGRE2 (EMR2) and rat ADGRL4 (15, 16).

There is a backlog demand for fundamental research on the aGPCR class, as the majority of members are still considered orphan receptors. However, there is an evident involvement of aGPCRs in various developmental and physiological processes and inherited diseases (17). Members of the aGPCR class often stand out with extraordinary long ECRs, which comprise module-like domain arrangements and many glycans. The so-called pentraxin (PTX)-like domain is one of these extracellular domains. Sequence homology predicts PTX-like domains to be present within the ECRs of the aGPCR members ADGRD1 (GPR133), ADGRD2 (GPR144), ADGRG4 (GPR112), and ADGRG6 (GPR126) (6). However, knowledge is lacking about the function of these domains within the ECRs of aGPCRs. The term “pentraxin” is inevitably linked to the homopentamerization of the archetypal serum proteins C-reactive protein (CRP) and serum amyloid component P (SAP). Indeed, pentamerization and other oligomeric states beyond dimers have been reported for other PTXs (18, 19). Therefore, the question arises whether PTX-like domains in ECRs of aGPCRs oligomerize in a similar fashion as their soluble counterparts. Furthermore, CRP and SAP feature distinct Ca^2+^-binding sites that are mandatory for ligand binding and, in case of CRP, for pentamerization (20, 21). Despite being distinct protein classes, the basic β-sandwich fold of the PTXs is also shared by other domain classes. Specifically, the laminin G–like (LG) domains exhibit a high structural resemblance to PTXs, despite a low sequence similarity. A more distant structural homology is also found for legume lectins, galectins, and bacterial β-glucanases (22). The laminin α subunit possesses five LG domains at its C terminus (LG1–5), which contain the major adhesive sites of laminins in the basal membrane (23, 24). After discovery in the laminin α subunit (25), LG domains were identified in other proteins of diverse biological function (22). Their function was linked to, for example, α-dystroglycan binding and similar binding modes were suggested for the LG3 domains of the heparan sulfate proteoglycans agrin (26) and perlecan (27). In neurexins, LG domains mediate a Ca^2+^-dependent interaction with neuroligin (28), forming a synaptic adhesion complex. LG domains were identified in the sex-hormone binding globulin (SHBG), which represents a soluble steroid hormone transporter protein (29). This multifaceted interaction spectrum of LG domains led to their alternative naming “laminin-neurexin-SHBG” domains (30).

ADGRG4 is an orphan aGPCR that was identified as a specific marker for enterochromaffin cells of the small intestine and neuroendocrine carcinoma cells (31). ADGRG4 is an evolutionarily old receptor being present in the genomes of all bony vertebrate classes (32). Among aGPCRs, ADGRG4 is a poorly studied GPCR and limited information is available about its physiological relevance yet. Recently, the 7TM domain of ADGRG4 has been structurally characterized using cryo-EM (33) displaying the same tethered *Stachel* peptide-mediated activation mechanism as for other aGPCRs (34). With more than 2700 amino acid residues in its ECR, this receptor presents with one of the largest N termini within the aGPCR class. However, only three domains are predicted by homology within this huge ECR—the PTX-like domain, the hormone receptor binding motif (HRM), and the GPCR autoproteolysis-inducing (GAIN) domain (Fig. 1*A*). The structural analysis of this ECR may provide clues to ADGRG4 function. GAIN and HRM domains of several other aGPCRs have been already structurally characterized by X-ray crystallography (35, 36, 37). Because of its peculiar architecture as a highly conserved domain at the tip of a huge probably disordered region, we were interested in the structure of the PTX-like domain (G4-PTX) to understand its relevance in ADGRG4 function. Our X-ray crystallographic studies of G4-PTX revealed an overall fold similar to those of other PTX domains. However, G4-PTX homodimerizes Ca^2+^ independently, as also supported by small angle X-ray scattering (SAXS) measurements, and significantly differs in distinct structural features compared to known PTX structures. Our data strongly suggests that ADGRG4 may act as a mechanical force sensor for shear stress or changes in distance.

**Figure 1:**
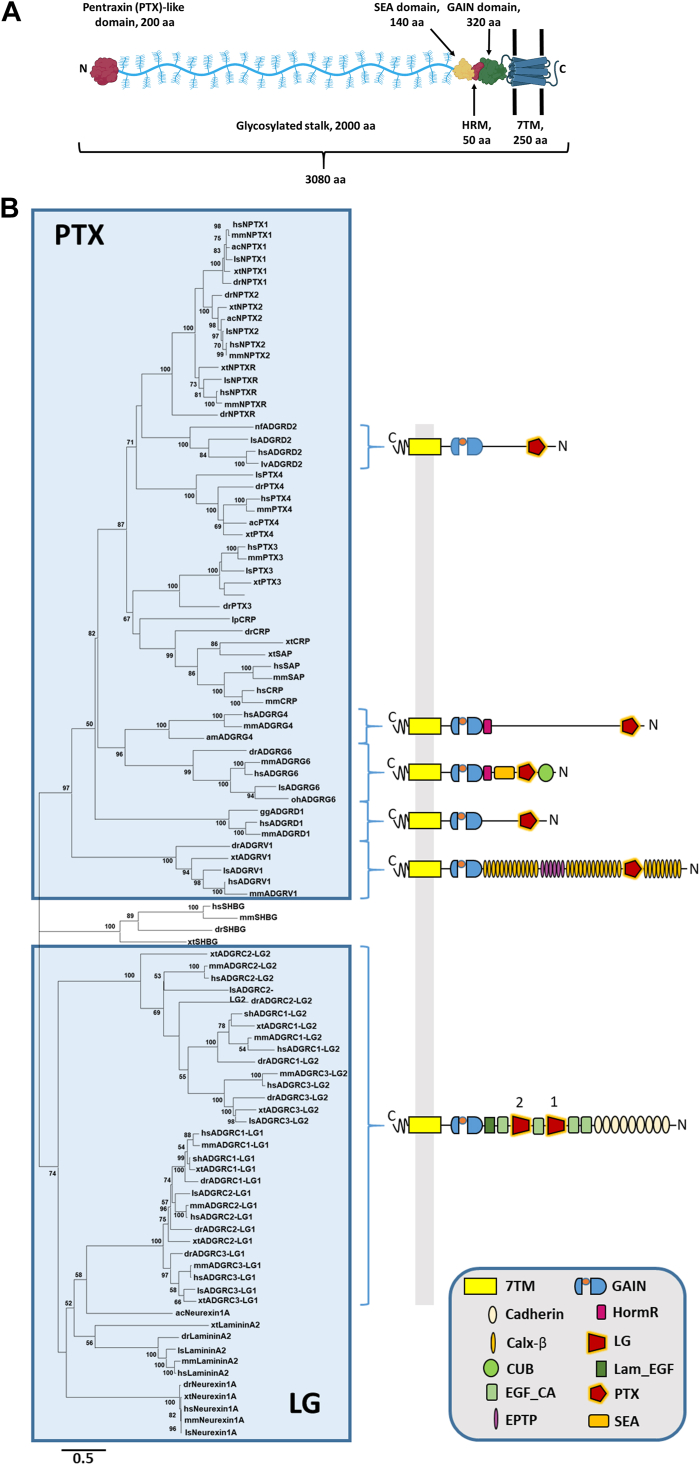
**Predicted molecular architecture of ADGRG4 and phylogenetic relation of PTX-like domains.***A*, the structural organization of the human ADGRG4 is shown; predicted domains are indicated with their rough amino acid lengths. Created with BioRender.com. *B*, phylogenetic tree of selected PTX-like domains was generated by the maximum likelihood method (see Experimental procedures). Species ac: *Anolis carolinensis* (*green* anole), am: *Alligator mississipiensis* (American alligator), dr: *Danio rerio* (zebrafish), gg: *Gorilla gorilla*, hs: *Homo sapiens*, lp: *Limulus polyphemus* (Atlantic horseshoe crab), ls: *Lonchura striata domestica* (Bengalese finch), lv: *Lipotes vexillifer* (Chinese river dolphin), mm: *Mus musculus* (house mouse), nf: *Nothobranchius furzeri* (turquoise killifish), oh: *Ophiophagus hannah* (king cobra), sh: *Strigops habroptila* (kakapo), xt: *Xenopus tropicalis* (western clawed frog). PTX, pentraxin.

## Results and discussion

### Phylogenetic analysis of aGPCR PTX-like and laminin-like domains reveals two distinct clusters

There are several PTX-like and LG domains in different soluble and membrane proteins. First, we analyzed their phylogenetic relations to G4-PTX by aligning selected orthologous amino acid sequences of PTX-like/LG domains from different proteins and building a maximum likelihood tree. As shown in Figure 1*B*, there are three main clusters as supported by bootstrap analysis—cluster “PTX” contains the PTX-like domains of the neuronal PTXs NP1 and 2, the neuronal PTX receptor, CRP, SAP, PTX3, PTX4, as well as the aGPCRs ADGRD1, ADGRD2, ADGRG4, and ADGRG6. Cluster “LG” comprises the LG/PTX-like domains of neurexin 1α, laminin α LG2, and the LG1 and 2 domains of the aGPCRs ADGRC1–3. The third cluster lies in between the two other clusters and is exclusively formed by SHBG orthologs. Within the cluster "PTX," the PTX-like domain of ADGRG4 clusters with those of ADGRG6, indicating close phylogenetic relation. The PTX-like domain of CRP and SAP, however, seems to be more distantly related to one of ADGRG4, already indicating some significant structural differences to the prototypical domain. Interestingly, the two LG domains of the ADGRC1–3 independently cluster according to their position within the ECR, regardless of their paralog affiliation. This suggests that the two different LG repeats in ADGRC1–3 fulfill distinct functions.

### The N terminus of ADGRG4 represents a conserved β-sandwich fold

**Figure 2:**
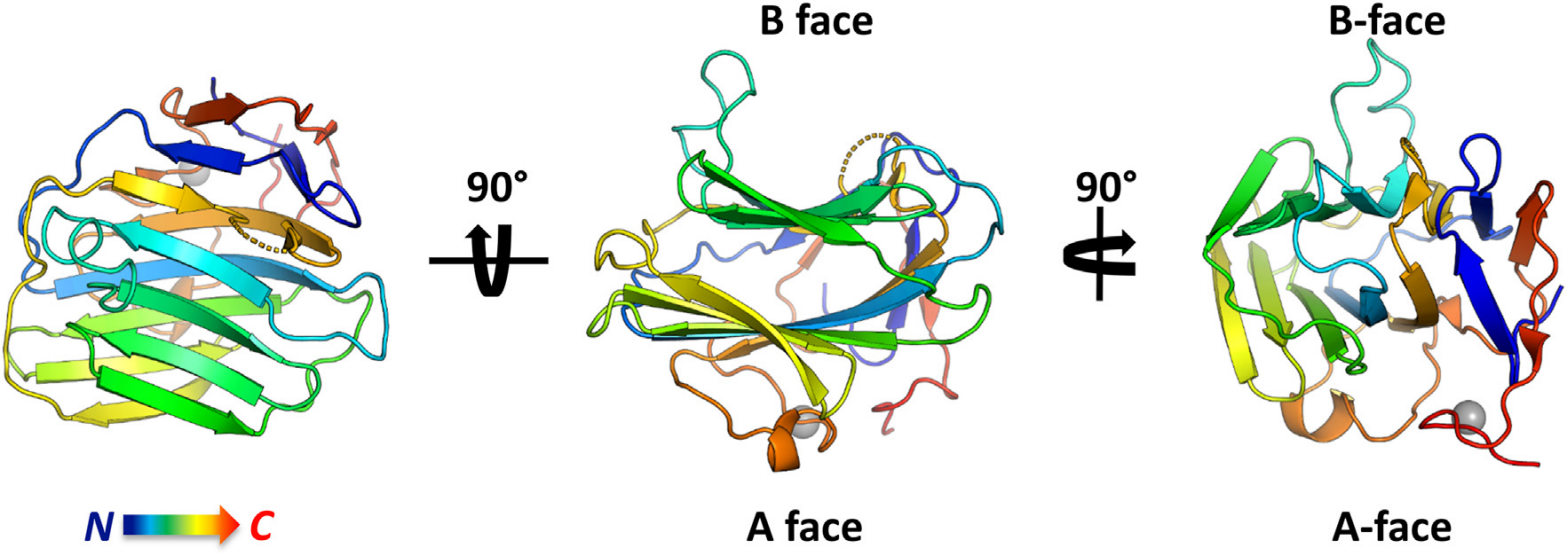
**Tertiary structure of G4-PTX in a rainbow-colored cartoon representation.** The *gray* sphere represents a bound Mg^2+^ ion. PTX, pentraxin.

**Figure 3:**
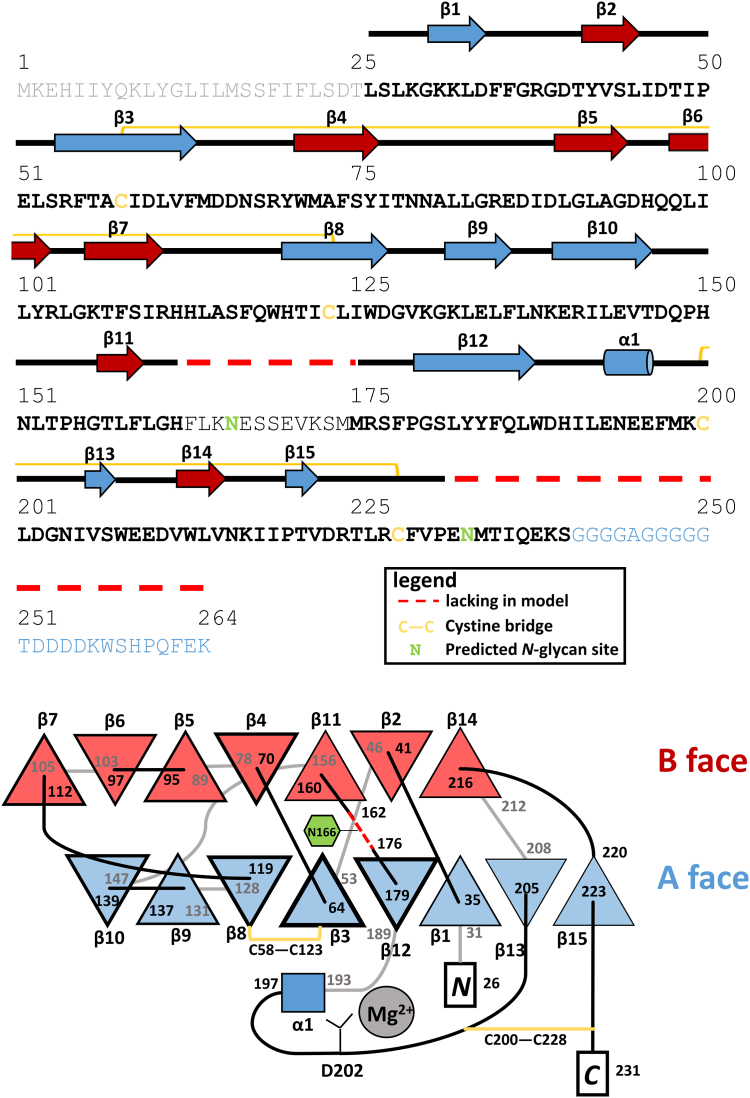
**G4-PTX secondary structure (*top*) and topology diagram (*bottom*).***Bottom* scheme: *Black*/*gray* lines depict loops in front/rear of the panel plane. The nomenclature of the β-sheets as A face and B face is made according to the structural relatives CRP and SAP. Frame thickness of the β-strands correlates with their respective aa length. Note that this scheme is not drawn to scale. CRP, C-reactive protein; PTX, pentraxin; SAP, serum amyloid component P.

For protein crystallization and structural analysis, the complementary DNA of the PTX-like domain of the human ADGRG4 (residues 1–240, UniProt accession number Q8IZF6-1; Fig. S1) was cloned into a mammalian expression vector and transfected for secreted expression into the HEK293S GnTI^-^ cell line. A two-step purification protocol (see Experimental procedures) yielded the protein in sufficient purity for crystallization (Fig. S2*A*). The protein exhibited a double band in a 12%-SDS-PAGE, which collapsed into a single band upon treatment with PNGase (Fig. S1*B*), indicating a heterogeneous glycosylation state. Optimized crystals had a size of over 200 μm. (Fig. S1*C*). Residues 26 to 162 und 176 to 231 are resolved in the electron density maps. The N-terminal residues 1 to 25 are a signal peptide and are not present in the secreted protein. The C-terminal residues as well as the loop residues 163 to 175 have no density, most likely due to flexibility. The crystallographic data revealed a β-sandwich fold (Fig. 2). Six and seven antiparallel β-strands assemble the two β-sheets A and B, respectively. The designation as "A" and "B" was adopted from the homologous PTX domains of CRP and SAP (Fig. 1*B*) (20, 38). The left panel of Figure 2 represents a top view onto the B face. This concave side of the β-sandwich features two protruding loops formed by residues 78 to 89 and 160 to 179 (Fig. 3), with the latter one being mostly unresolved in the electron density map. The A face is covered by another loop of residues 189 to 205 with a short helical stretch. This loop coordinates a metal ion *via* the side chain carboxy group of the residue D202. This metal ion was modeled as Mg^2+^ based on an average coordination distance of 2.0 Å and the presence of 0.2 M MgCl_2_ in the crystallization medium (Figs. 2 and S3*A*). The octahedral coordination sphere of the magnesium ion is completed by five water molecules, which in turn are not directly bound by other parts of the protein.

Two disulfide bridges stabilize the protein fold (Fig. S3*B*). C58-C123 links strands β3 and β8 in the β-sheet of the A face (Fig. 3). C200-C228 connects the long A face loop 189-205 with the C-terminal residues of the PTX-like domain. This domain contains two putative N-glycosylation sites at N166 and N233, with the latter being more likely to be glycosylated according to predictions of the NetNGlyc server (39). The presence of N-glycosylation was verified by SDS-PAGE–based glycan staining (Fig. S2). Due to the disorder of residues 163 to 175 and thus their absence in the electron density, however, we do not have experimental evidence for glycosylation at this site.

**Figure 4:**
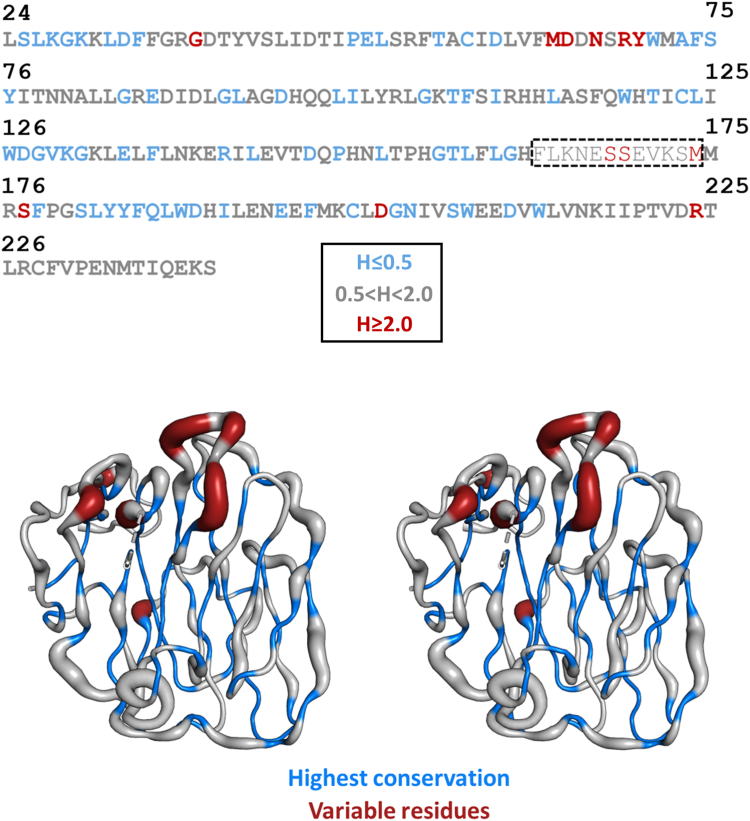
**Shannon entropy based on multiple sequence alignment of 59 mammalian species plotted onto the structural model.** View onto the G4-PTX B face in wall-eyed stereo display. The model shows the C_α_-trace in differing thickness. The *thickness* represents the Shannon entropy, H, of a given position and thus its evolutionary variability. Residues of low variability are additionally highlighted in *blue*, variable residues are shown in *red*. In general, a residue exhibiting H<2.0 is considered conserved (93, 98). The unresolved loop is marked in the sequence by a *dashed* box. PTX, pentraxin.

A multiple amino acid sequence alignment of 59 mammalian ADGRG4 orthologs was employed to determine the Shannon entropy (40) as a measure of evolutionary conservation for each residue of the PTX domain (see supplementary data). The result mapped onto the G4-PTX structure is shown in Figure 4. Overall, there is a high degree of amino acid sequence conservation among the species, even for most surface residues. Especially the core strand residues of the β-sandwich exhibit the lowest Shannon entropy and thus highest conservation. The highest evolutional variability is found for the loop 64-70 connecting strands β3 and 4 and the unresolved loop 160-179. Longer stretches of extraordinary high conservation comprise the N terminus including β1, β4, β8, and β9 including their connecting loop, β11 and β12. Interestingly, these conserved sections represent pairs of antiparallel β-strands: β1- β12, β8-β9 (A face), and β4-β11 (B face).

### The PTX-like fold exhibits distinct differences to structural homologs

**Figure 5:**
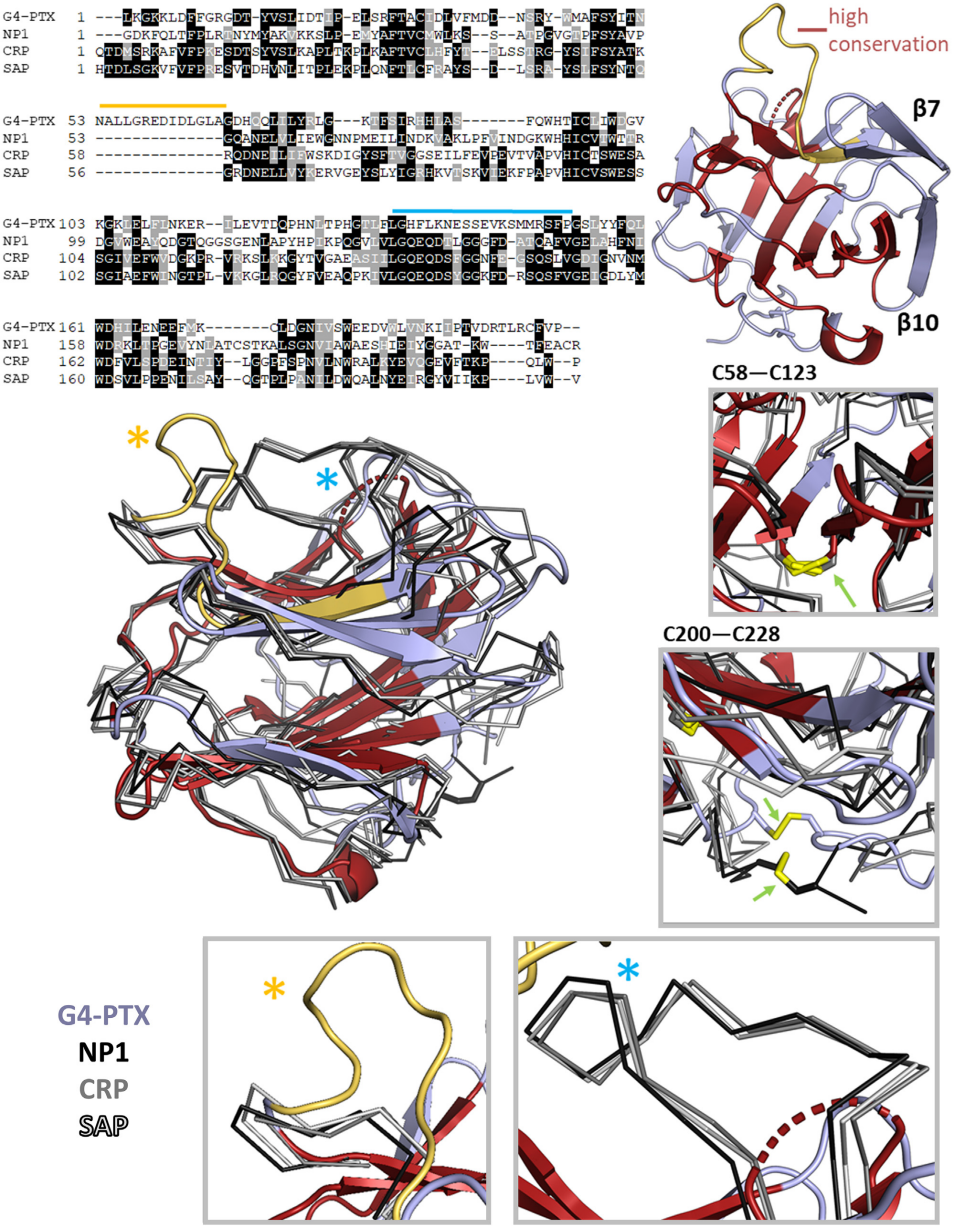
**Alignment with closest structural homologs.** Shown is the aa range of G4-PTX 26-231, NP1 (6ype (42)) 225-429, CRP (1b09 (99)) 19-224, and SAP (2a3y (100)) 20-223. Regions of the highest sequence consensus (based on the TCoffee server (101)) are highlighted in *red* in the G4-PTX structure. *Boxes* showcase distinct features of G4-PTX in comparison to its structural relatives. Note that disulfide bridge C58-C123 is present in all four structures, whereas disulfide bridge C200-C228 is only found in G4-PTX and NP1. The box with the *yellow asterisk* shows an extended loop which is solely present in G4-PTX. The box with the *blue asterisk* depicts a close-up view on the long loop of the B face, which exists in all structures, but is unresolved (flexible) in the G4-PTX structure. CRP, C-reactive protein; PTX, pentraxin; SAP, serum amyloid component P.

To identify structural similarities, the refined structural model of G4-PTX was submitted to the PDBeFold server for pairwise structural alignment with existing PDB structures (41). Based on the r.m.s.d. of the C_α_-chains, a selection of different structural relatives is given by Table S1. The closest structural relation is found for the human neuronal PTX 1 (NP1, 25% identity) (42). The structures of the human CRP and human SAP follow very closely in this structural comparison. Figure 5 shows a multiple sequence alignment (MSA) of the four proteins. The majority of the A face β-strands shows high conservation, in contrast to the B face being less conserved among the regarded PTXs. Loops and outer β-strands are generally more variable. Interestingly, the disulfide bridge C58-C123 (G4-PTX) that links two core β-strands of the A face is conserved among all structural relatives. However, the second disulfide bridge C200-C228 is not analogously found in CRP and SAP, but it is present in the NP1 structure. Furthermore, the amino acid sequence alignment indicates a G4-PTX section, which does not align to the other sequences (marked yellow in Fig. 5). It forms the extended B face loop 53-66, which is not present in the related structures. The second B face loop 160-179 (marked blue in Fig. 5) is partially shared by all four PTXs. The loop sequence of G4-PTX, however, is mostly unrelated to its structural homologs and it is partially disordered. In contrast, the other PTXs share a high sequence identity and structural similarity in this area.

### The N terminus of ADGRG4 shows Ca^2+^-independent homodimerization

**Figure 6:**
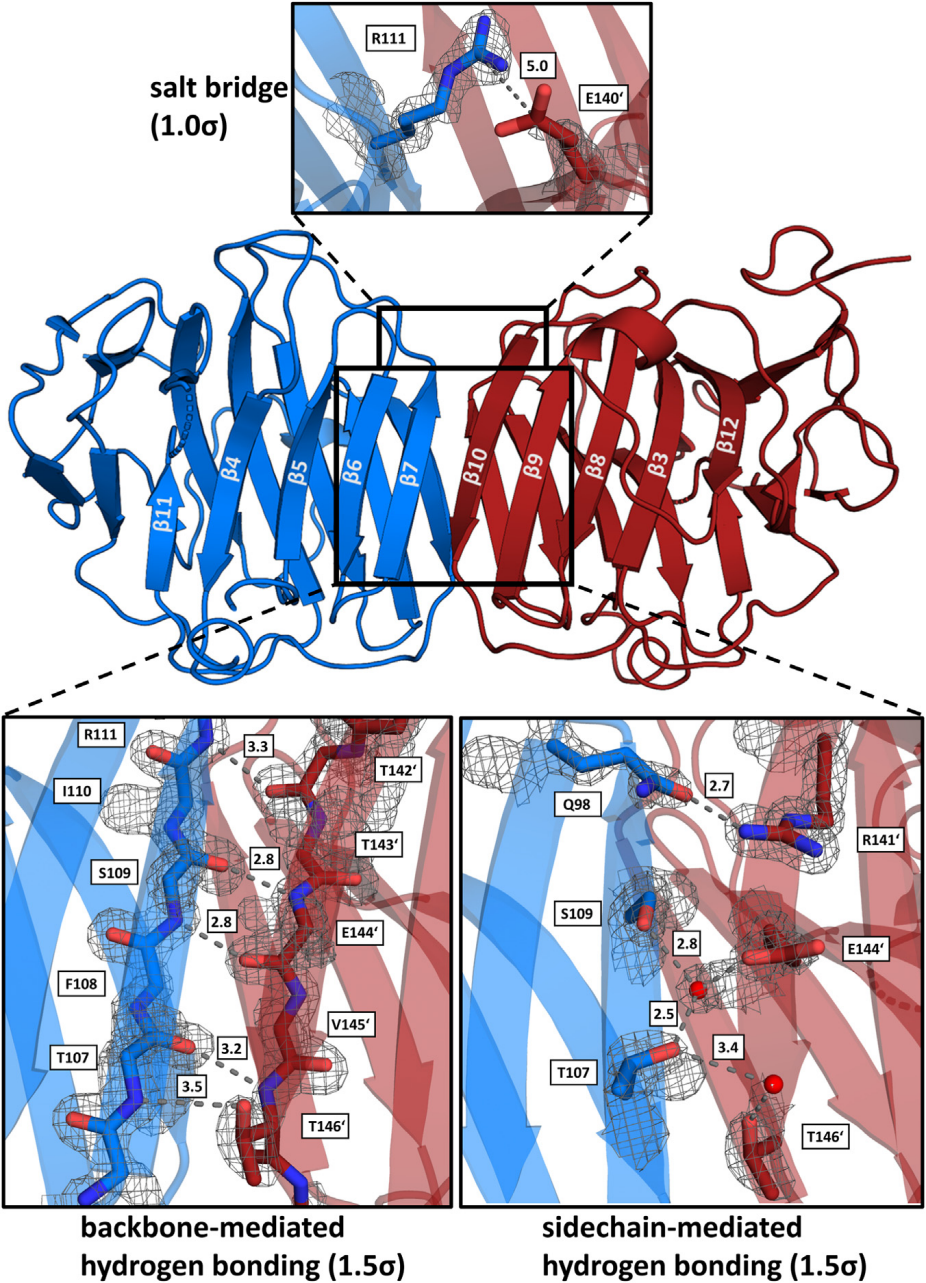
**Interactions at the homodimer interface.** For a better overview, backbone-mediated hydrogen bonds are shown separated from side chain–only interactions. Distances are stated in Å next to the dashed connectors. The contour levels of the (2F_o_-F_c_)-type electron density map are given next to the images.

From dynamic light scattering (DLS) experiments of G4-PTX, a hydrodynamic radius was calculated that was too large for an expected monomer mass of 30.5 kDa. To obtain more precise information about the molar mass in solution, static light scattering (SLS) was performed (Fig. S4). Indeed, the molecular mass determined by SLS increased with protein concentration and indicated a monomer-dimer-equilibrium in solution. The unit cell of the G4-PTX crystals on the other hand contained only a single protein chain in the asymmetric unit. To address a potential homodimer interface, the respective symmetry mates in the C2 space group were assessed. Four different types of protein-protein contacts are present within the crystal lattice (Fig. S5*A*). However, there is only one interface generated by point symmetry that exhibits a sufficient surface area for homodimerization. Figure 6 gives an overview of the interactions that constitute this dimer interface. Two protomers interact such that the two strands at the edge of the sandwiched β-sheet interact with those of the other molecule (β7 with β10′ and β10 with β7′, Figs. 3 and 6). By this interaction, two large continuous β-sheets are formed across the dimer interface. Core interactions are mediated by peptide backbone hydrogen bonds between each pair of opposing antiparallel strands (Fig. 6) and by the apolar side chains enclosed by the sandwiched β-strands 7 and 10, which have a tight steric fit and therefore exert strong hydrophobic interactions. The peptide backbone hydrogen bonds are furthermore supported by several side chain hydrogen bonds. Here, the tightest interaction is formed between the carboxamide-O_ε_ of Q98 from β-strand 6 and the guanidinium moiety of R141′ of β10 (Fig. 6). Additional hydrogen bonds are established between residues T107-T146, T107-E144 (*via* a water molecule), and S109-E144 (*via* a water molecule, Fig. 6). An ionic interaction is possible between R111 and E140′ at the interface flanks; however, for both residues, the density indicates significant side chain flexibility.

To further characterize the monomer-dimer-equilibrium, SAXS experiments were performed at different AGDRG4-PTX concentrations. The scattering profiles exhibited concentration-dependent changes, which are highlighted by the observed R_g_ (radius of gyration) and D_max_ (maximum diameter of particle) values at different G4-PTX concentrations (Table 1).

**Table 1:** Basic SAXS parameters of a G4-PTX concentration series as obtained *via* PRIMUS

c(G4-PTX)/mg mL^−1^	Guinier approximation data range/qR_g_ range	P(r)-function data range	Guinier R_g_ (real-space)/Å	Guinier I(0) (real-space)/cm^−1^ ml mg^−1^	D_max_/Å	V_p_/Å^3^	MW/Da	Range of calculated R_g_ values of NNLSJOE ensemble models for monomer/dimer [Å]	SASBDB accession code
0.27	20–168/0.20–1.30	20–1071	26.8 ± 0.4 (25.7)	0.022900 ± 0.000170 (0.02)	71.3	67,079.3	41,276	22.8–27.6/29.8–35.3	SASDSE8
0.55	25–162/0.25–1.30	25–1035	27.7 ± 0.2 (27.1)	0.026000 ± 0.000100 (0.03)	80.3	65,094.5	47,835	23.3–27.6/31.5–33.0	SASDSF8
1.07	27–153/0.27–1.28	27–995	28.8 ± 0.1 (28.8)	0.028900 ± 0.000061 (0.03)	89.9	70,094.0	47,162	23.3–23.8/32.1–33.0	SASDSG8
1.88	27–150/0.28–1.29	27–968	29.6 ± 0.1 (30.1)	0.029100 ± 0.000038 (0.03)	96.8	73,516.7	50,944	23.3–23.4/31.0–33.1	SASDSH8
4.37	28–145/0.29–1.26	28–958	29.9 ± 0.1 (30.5)	0.033300 ± 0.000029 (0.03)	99.2	76,387.9	51,600	23.0–23.3/30.3–32.7	SASDSJ8
6.38	30–147/0.31–1.27	30–961	29.8 ± 0.0 (30.4)	0.033200 ± 0.000019 (0.03)	99.0	77,097.5	55,527	23.0–23.3/30.3–32.7	SASDSK8
7.51	59–152/0.54–1.30	59–975	29.4 ± 0.0 (30.0)	0.030600 ± 0.000018 (0.03)	96.2	77,004.4	55,597	23.3/30.3–32.7	SASDSL8
9.15	19–150/0.21–1.28	19–978	29.3 ± 0.0 (30.0)	0.030800 ± 0.000011 (0.03)	107.7	78,302.6	57,592	23.3–24.0/30.3–32.7	SASDSM8

The Guinier plots were optimized by AUTORG and the pair-distance distribution function was obtained by AUTOGNOM. Molecular weights were approximated using the “Size and Shape” option in PRIMUS of the ATSAS suite. R_g_ denotes the radius of gyration, I(0) denotes the extrapolated intensity at Θ = 0°, D_max_ denotes the maximum extension of the particle, V_p_ denotes the Porod volume, and MW denotes the molecular mass. The calculated R_g_ values of the monomer are 21.9 to 22.5 Å and of the dimer 29.5 to 30.38 Å, depending on the glycosylation state.

We first calculated the fit of the four potential dimer structures observed in the crystal structure to the experimental SAXS data (program OLIGOMER). The favored homodimer structure with C_2_ symmetry exhibited the best fit with χ^2^ = 1.36 (Fig. S5*B*).

**Figure 7:**
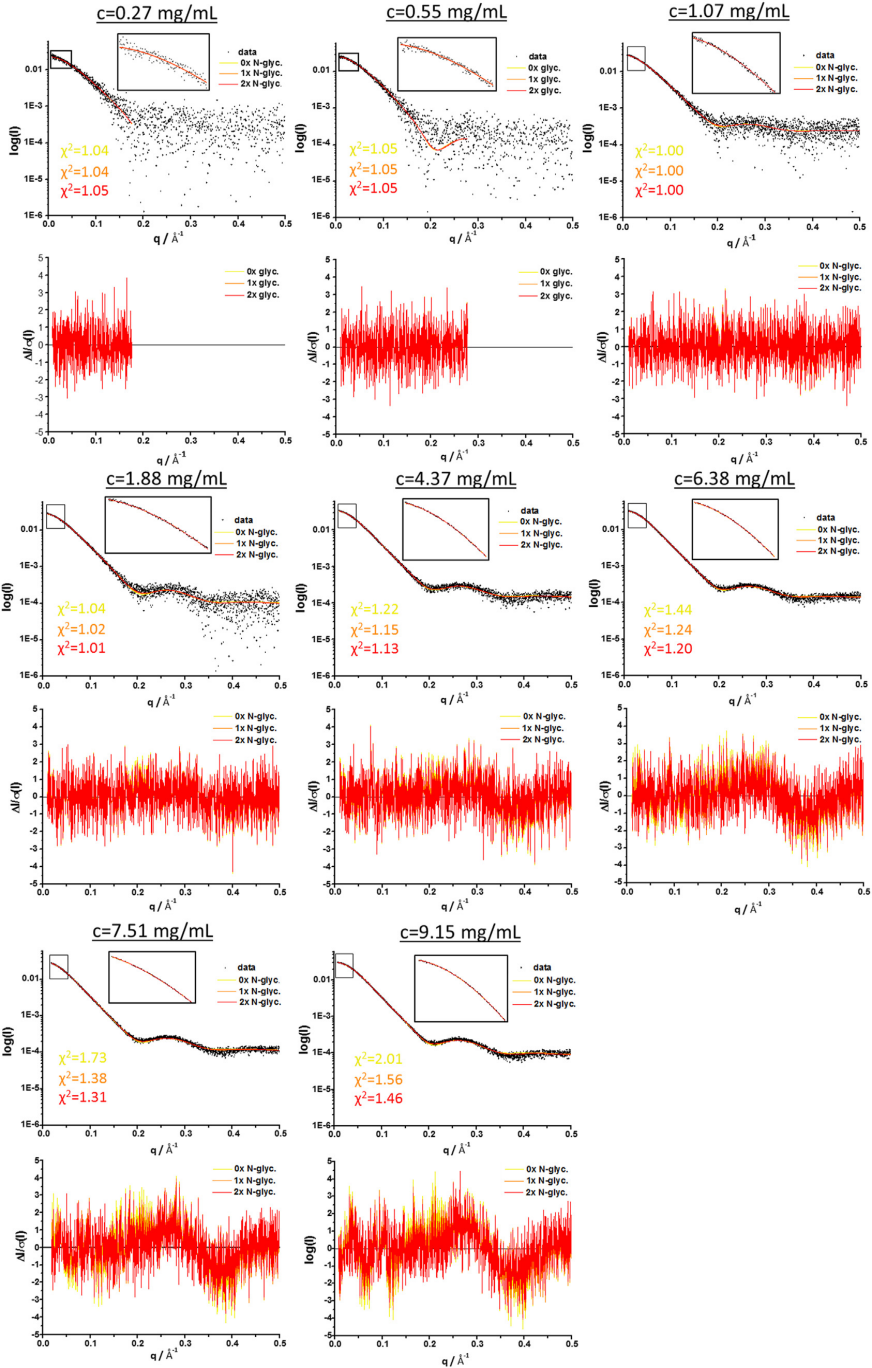
**EOM fits to SAXS data.** SAXS curves at different G4-PTX concentrations are shown with their respective fit as obtained from EOM using the NNLSJOE algorithm. Three different fits represent the three different model pools that were comparatively employed (zero to two times N-glycosylated G4-PTX). Boxes zoom into the low q-region of the plots, each ranging until q ≤ 0.05 Å^−1^. Normalized residual plots are depicted for each fit. For the first two datasets of very dilute samples (0.27 mg/ml and 0.55 mg/ml), the ATSAS suite program SHANUM (94) was employed to determine the useful data range and avoid overfitting of the noisy high q-region. PTX, pentraxin; SAXS, small angle X-ray scattering.

**Figure 8:**
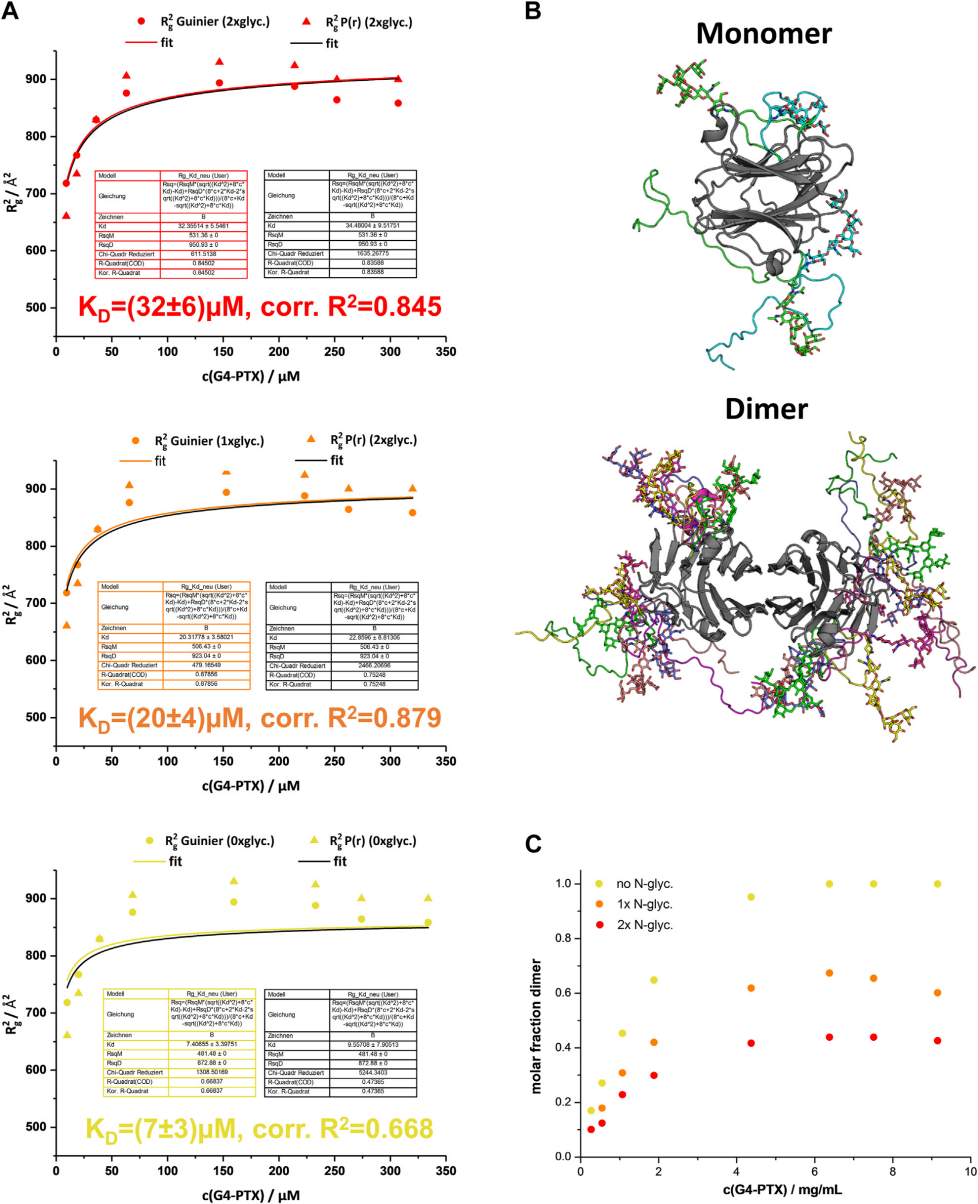
**SAXS-data analysis.***A*, plot of R_g_^2^*versus* c(G4-PTX). The R_g_ values were derived from Guinier approximation and the P(r)-function. K_D_ values were determined by nonlinear regression *via* Equation (4). Each diagram shows fits derived for different theoretical R_g_ values of monomer and dimer depending on the glycosylation state (zero to two N-glycosylations). The resulting K_D_ values based on the Guinier R_g_ are specified in each diagram. *B*, representative examples of fully N-glycosylated monomer and dimer models selected by EOM at a G4-PTX concentration of 1.88 mg/ml. This ensemble consists of two monomers and five dimers (each with different glycan conformations). *C*, molar fractions of homodimer obtained by EOM analysis plotted against G4-PTX concentration. The molar fractions are calculated for each ensemble generated for the three different model pools, differing in N-glycosylation. PTX, pentraxin; SAXS, small angle X-ray scattering.

Next, we determined the molar fractions of monomer and dimer at different protein concentrations *via* ensemble optimization modeling with the program EOM (Fig. 7) (43, 44). To assess the influence of G4-PTX glycosylation on the scattering, three different model pools were employed, featuring a different degree of N-glycosylation (no, one, or two N-glycosylation sites). However, there are only minor differences notable in the quality of EOM fits. The fully N-glycosylated model pool generated the best fitting ensembles. In a second fitting strategy, we first generated theoretical scattering curves for the monomer pool and for the dimer pool, separately for the three glycosylation states. Next, the two curves were fit to the experimental data *via* linear combination using OLIGOMER (45) (Figs. S8 and S9). In general, the EOM ensembles resulted in better fits compared to the OLIGOMER fitting strategy, especially for the data obtained at higher protein concentrations. Figure 8*C* shows a plot of the molar fraction of G4-PTX homodimer *versus* protein concentration. These curves show concentration-dependent dimer formation which reaches saturation at comparable concentrations. However, only the ensemble with nonglycosylated models reaches a dimer fraction of 100% at 6.38 mg/ml, whereas the fully glycosylated models resulted in a selection of only ∼40% homodimer models at this protein concentration. This phenomenon was mostly independent of the q data range and observed for different fitting strategies of EOM (NNLSJOE or GAJOE) and also for the OLIGOMER fit using the combined monomer and dimer pools. A possible cause is that the generated models of the disordered loops and glycans do not represent the conformational flexibility of these regions sufficiently. As a result, the monomeric fraction is overestimated in the fitted ensembles.

On the other hand, the experimental R_g_ values determined by Guinier approximation are in good agreement with full homodimer formation (Fig. S6). At c = 6.38 mg/ml, an R_g_ of 30.4 Å was obtained (Table 1). Given an average theoretical R_g_ of 30.8 Å for the pool of glycosylated dimer models and 23.1 Å for the monomeric pool, 88% of homodimer should be present in solution (see Equation (1)).

To quantify the affinity of G4-PTX homodimer formation, we performed nonlinear regressions on both the experimental R_g_^2^ *versus* concentration plots (Fig. 8*A*) as well as the x_D_ (dimer mol fraction) *versus* concentration plots obtained by ensemble modeling (Fig. S7). K_D_ values obtained from the experimental R_g_^2^ plots range between 7 and 32 μM, depending on the presumed glycosylation state. The fits to the NNLSJOE x_D_ *versus* concentration plots resulted in K_D_ values of 8 to 45 μM.

**Figure 9:**
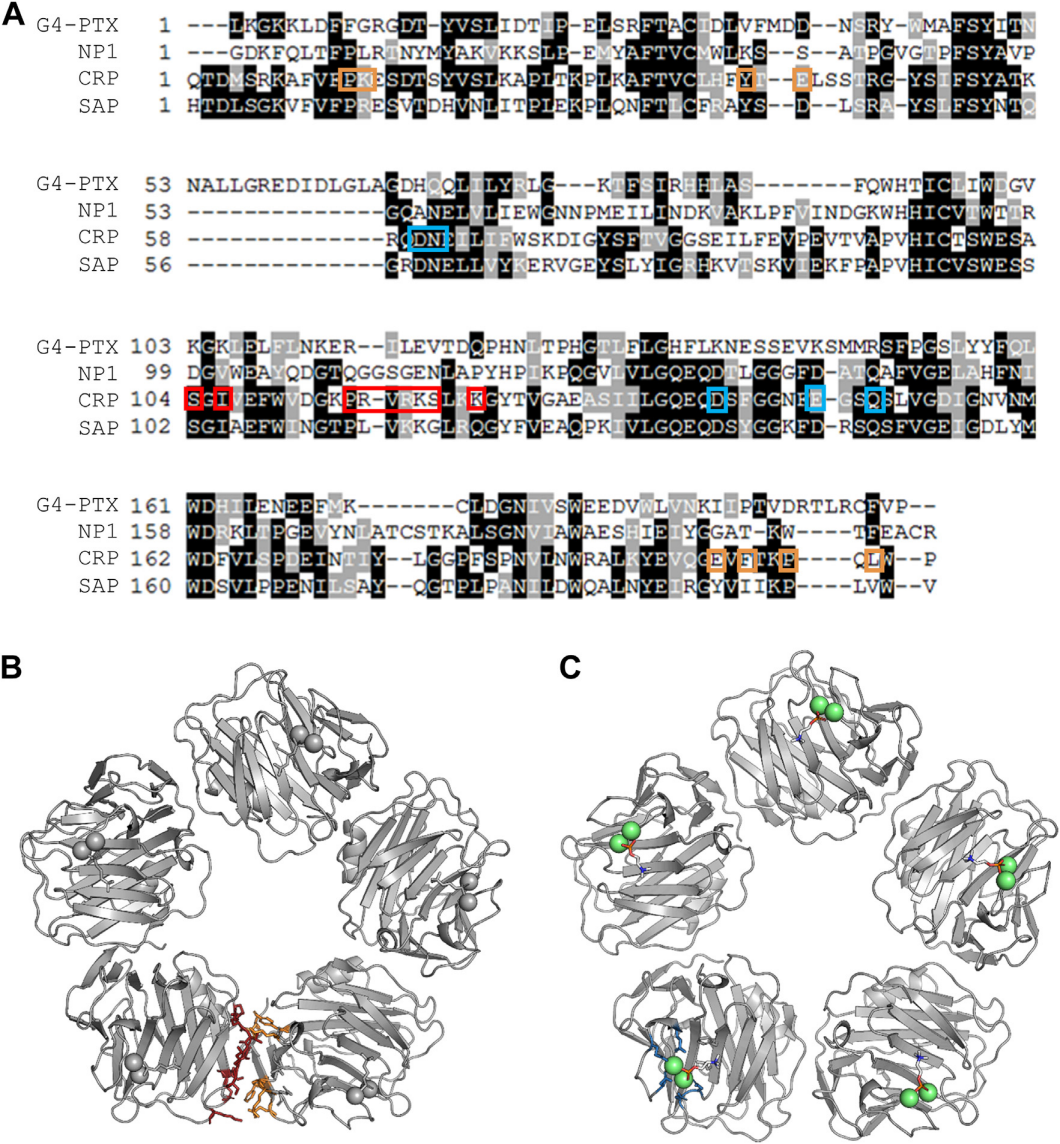
**CRP pentamer interfaces and Ca^2+^-binding sites.***A*, alignment of G4-PTX with structural homologs. The regions framed in *red* and *orange* correspond to CRP pentamer interfaces as indicated by these residues in the CRP pentamer in panel (*B*). Residues indicated in *blue* correspond to the Ca^2+^-binding site, as shown in panel (C). Note, there is no sequence similarity between G4-PTX and CRP in the highlighted regions. CRP, C-reactive protein; PTX, pentraxin.

The classical PTXs CRP and SAP feature characteristic Ca^2+^-binding sites, which are indispensable for ligand binding and, in case of CRP, pentamer stabilization (Fig. 9) (20). In the elucidated structure of G4-PTX, however, only one bound metal ion was detected (Figs. 2 and S3*A*), which is loosely coordinated by a single carboxylate side chain. A potential influence of physiological Ca^2+^ concentration on the protein's quaternary structure was assessed by DLS, SLS, differential scanning fluorimetry, and crystallography. No significant change in the hydrodynamic radii and molecular mass was observed in the presence of Ca^2+^ (Fig. S10*A*). However, CaCl_2_ addition increases the melting temperature T_m_ by 1.6 K, indicating a stabilizing effect (Fig. S10*B*). To study potential structural consequences of Ca^2+^ presence, crystals were soaked with 100 μM, 1 mM, or 10 mM CaCl_2_ for 1 h or 19 h. Furthermore, the protein was crystallized in the presence of 10 mM CaCl_2_. At least one dataset per condition was recorded and analyzed. However, none of these datasets revealed new features in the electron density compared to the previous results without Ca^2+^ addition (data not shown). Support for a Ca^2+^-independent dimerization comes also from the comparison of the pentamer interfaces and the Ca^2+^-binding sites of CRP with the G4-PTX structure (Fig. 9). The pentamer-forming interaction sites in CRP and the corresponding positions in G4-PTX show low conservation (Fig. S11). This makes a G4-PTX oligomer formation similar to CRP unlikely. Furthermore, the Ca^2+^-coordinating carboxyl groups in CRP are not conserved in G4-PTX (Fig. 9). These facts together with the distant phylogenetic relation between CRP and G4-PTX (Fig. 1*B*) suggest that the PTX domain does not form Ca^2+^-dependent pentameric homophilic interactions.

As described before, the G4-PTX dimer interface is mainly formed by interactions between strands β7 und β10 at the edge of the central β-sheets. Since the hydrophobic side chains between these sandwiched β-strands are relatively well-conserved for their structural function in the densely packed core of the fold, it is difficult to deduce an additional functional role in dimer formation by conservation analysis (as opposed to generally less-conserved solvent-exposed surface residues). We therefore analyzed the prediction of dimer structures by the AlphaFold machine learning algorithms (46). AlphaFold uses two main sources of structural information for its prediction: (i) experimentally determined template structures of related proteins and (ii) coevolution of residues in spatial proximity in the folded protein. The first ranked dimer model matches the experimental G4-PTX dimer structure well (Fig. S12). Also, the model confidence of the dimer structure is high as demonstrated by the low predicted aligned errors between the protomers (PAE parameter in Fig. S12). As the template structures involved in modeling the dimer structure did not include structures that resemble the G4-PTX dimer, the model is predominantly based on information from sequence coevolution. Next, we also predicted the dimer structure for an additional 12 out of the 59 mammalian ADGRG4 homologs of the MSA (Figs. 4 and S13). The sequences were chosen to evenly cover the phylogenetic tree. This analysis indicates that formation of this dimer is a conserved feature for all mammals and that it has functional relevance for the receptor (see figure legend of Fig. S13 for further details).

### The putative dimer interface resembles that of SHBG and lectins

A very similar dimerization mode as found for G4-PTX has been described for the SHBG N-terminal LG domain (29, 47). Here, the N-terminal LG domain provides both the steroid-binding site and the homodimer interface. However, this dimerization is Ca^2+^-dependent and facilitated by ligand binding (48).

**Figure 10:**
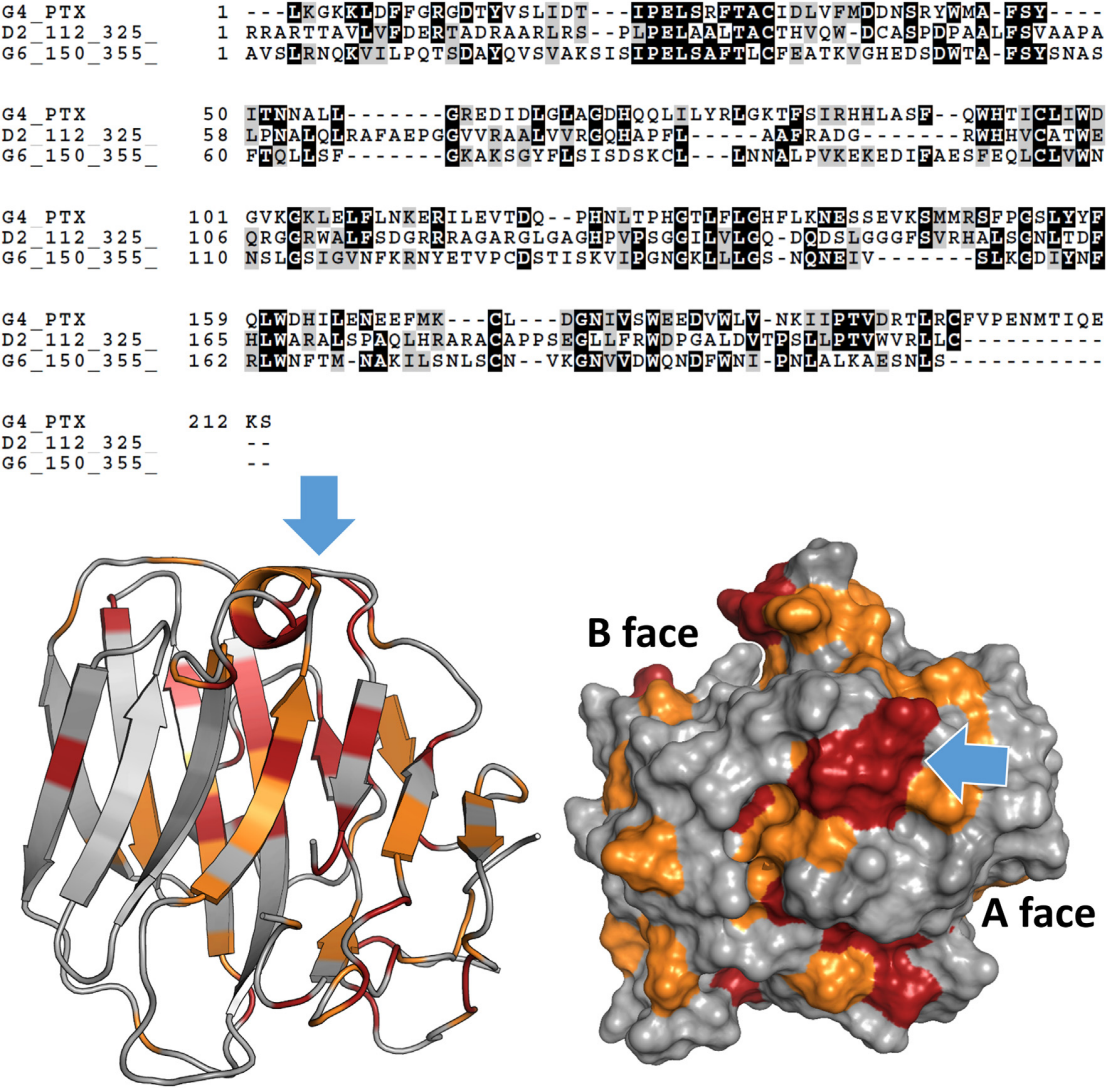
**Alignment of G4-PTX with homologous PTX domains of the human aGPCR members ADGRG6 (UniProt Accession Number: Q86SQ4-1), ADGRD1 (Q6QNK2-1), ADGRD2 (Q7Z7M1-1).** A *blue* box highlights a conserved patch of surface residues. *Bottom*: G4-PTX (view onto B face) with highlighted residues of high (*red*) and medium (*orange*) sequence consensus. *Blue arrows* indicate a conserved patch at the flank of the structure with a core “PEL” motif. aGPCR, adhesion G protein–coupled receptor; PTX, pentraxin.

Recently, Leon *et al.* (36) elucidated the structure of the zebrafish ADGRG6-ECR, which contains an internal PTX-like domain. However, the authors did not comment on the oligomeric state of the ECR in solution and the crystal structure does not feature a comparable dimer interface (PDB 6V55). As the ADGRG6-PTX domain represents the closest relative to G4-PTX, we also investigated ADGRG6 oligomerization. Therefore, the ECR of ADGRG6 was expressed in HEK293 cells, purified, and analyzed by SEC-MALS-SAXS coupling (to be published). Conversely, the obtained molecular mass matches perfectly the expected one of a monomeric ECR. Furthermore, there is only poor sequence homology between G4-PTX and ADGRG6-PTX within the two strands β7 and β10 that form the putative homodimer interface (compare Figs. 3 and 10). AlphaFold predictions for the PTX-like domains of ADGRD1, ADGRD2, and ADGRG6 do not indicate dimer formation (Fig. S14). Hence, we conclude that our observations for G4-PTX are not generalizable for other PTX-like domains found in aGPCRs.

### The typical binding rim of LG domains shows high surface conservation in G4-PTX

Despite their phylogenetic proximity, G4-PTX differs significantly from classical PTXs. This finding is supported by the non-PTX-like features of this “PTX-like” domain: There is no apparent Ca^2+^-dependent pentamerization. In contrast to classical PTXs, the functionally important B face does not exhibit the typical Ca^2+^-binding site (Fig. 9). Instead, the respective loop 160–179 is highly disordered and of comparably low evolutionary conservation with almost no sequence similarities to CRP or SAP (Fig. 5). In contrast to their high structural similarity, the main ligand-binding interface of PTX and LG domains differs significantly. While PTXs bind their ligands *via* the B face of the β-sandwich and its characteristic Ca^2+^-binding sites, LG domains employ the β-sandwich rim opposite to the N and C terminus as interaction area. This binding site is mainly established by the loops connecting strands β2–3 and β10–11. Diversity in these loops enables a broad ligand spectrum, with the core β-sandwich providing a rather rigid scaffold (22). In adhesive interactions between LG domains and proteoglycans, a divalent cation is typically involved, which is only weakly coordinated by one or two residues of the LG domain. For example, the LG4 and 5 domains of the α-subunit of laminin-2 bind a glycan chain of α-dystroglycan mediated by bound Ca^2+^. A similar mechanism is proposed for the heparin proteoglycans agrin and perlecan (49). For the binding of steroid hormones to SHBG, the same β-sandwich rim area is essential again. In contrast to G4-PTX and other LG domains, in SHBG, the loop linking strands β10 and β11 shows high disorder, which is probably critical for steroids to intercalate the β-sheets (50). Intriguingly, an alignment of all PTX-like domains present in aGPCRs reveals a conserved patch of surface residues, located within the β2-3 loop around a “PEL” consensus sequence (Fig. 10). The central, highly conserved glutamate residue (E51 for ADGRG4) protrudes from the surface (Fig. S15). This finding suggests a common interaction interface for ADGR-PTX–like domains, which resembles that of the LG domain binding rim.

### Possible implication of the G4-PTX domain structure on *in vivo* functions

In principle, G4-PTX homodimerization can occur at the same cell (*cis*) or between different cells in close proximity (*trans*) expressing the receptor protein. Homophilic interactions in *trans* were previously shown for other aGPCRs. For example, ADGRC1 (CELSR1) undergoes homophilic *trans* interactions and concentrates at cell-cell contacts in adherens junctions, where it is involved in the planar-cell-polarity pathway required for neural plate bending (10). *Trans* dimerization was found also for the homologs ADGRC2 and 3 (11). Similarly, Flamingo, a structurally related aGPCR in the fly *Drosophila melanogaster*, is localized at cell-cell boundaries of *Drosophila* wing cells and engages in planar-cell-polarity signaling (51). In both cases, *trans* homodimer formation is mediated by repeats of cadherin domains within the ECR. ADGRG1 (GPR56) represents another example of a homophilic *trans* interaction of the ADGRG subfamily. Here, Paavola *et al.* (52) showed that the homophilic N-terminal interaction enhances ADGRG1-mediated RhoA activation.

Given the size of over 2700 amino acid residues of the ADGRG4 ECR, which is even larger than the ECRs of ADGRC, it is reasonable to assume homophilic *trans* interaction mediated by the N-terminal PTX-like domain. The highly glycosylated mucin-like stalk (53) between the PTX-like and HRM domain might act as a spacer to span intercellular clefts, enabling the G4-PTX to reach remote binding partners. An unfolded protein has a contour length of approximately 4 Å per residue (54), but the chain is highly flexible and forms mostly globular random coil structures. It has been shown for mucins that O-glycosylation increases the stiffness (persistence length) of the peptide core about 10-fold resulting in the formation of an extended structure, such that the radius of gyration increases almost 10-fold (55). With a pitch of 2.5 Å, as determined for mucins, a fully extended ECR chain of ADGRG4 may span a distance of more than 0.5 μm. Recently, Malaker *et al.* (56) developed an algorithm to detect mucin-like domains within the human proteome. The algorithm first identifies O-glycosylation sites *via* the NetOGlyc 4.0 server (57). Overlapping phosphorylation sites are subtracted from the total O-glycosylation sites as false positives. Next, based on four mucin-typical benchmarks, the algorithm determines a so-called “mucin score”. A mucin score of >2 represents a high confidence for the mucin domain assignment. Values between 1.5 and 1.2 represent a low confidence, while lower scores are not considered mucin-like. Analysis of ADGRG4 results in a mucin score of 3.4, which clearly indicates the mucin-like properties of the receptor. The algorithm detected a total of 212 mucin-domains, which arrange seamlessly starting at residue 279 until residue 2244 (Uniprot Q8IZF6). This region starts C-terminally to the PTX-like domain and ends roughly 140 amino acids N-terminal to the predicted HRM. Interestingly, ColabFold (58) predicted this ∼140 amino acid stretch to establish a Sperm protein, Enterokinase, and Agrin (SEA) domain (Fig. S16). SEA domains are a typical feature of many mucins: MUC1, MUC3, MUC12, MUC13, and MUC17 mucins all possess a single and MUC16 even multiple SEA domains (59). However, the typical autoproteolytic site G↓S[V/I]VV present in many mucin SEA domains is lacking for the ADGRG4 SEA domain. The presence of SEA domains has been reported already for three other aGPCR ECRs, namely ADGRF1 (GPR110), ADGRF5 (GPR116) (53, 60), and ADGRG6 (GPR126) (36). The homodimerization of ADGRG4’s N-terminal domain presented in this study strongly suggests a homophilic *cis* but also a *trans* dimerization (<1 μM distance) of this receptor. We determined a K_D_ value of ∼40 μM for the dimerization *in vitro* (Fig. 7*C*). This translates into a binding ΔG of −26 kJmol^−1^ at 37 °C. Interestingly, the dimer interface does not mask the typical LG domain binding rim. Therefore, additional ligands may bind to the G4-PTX homodimer.

**Figure 11:**
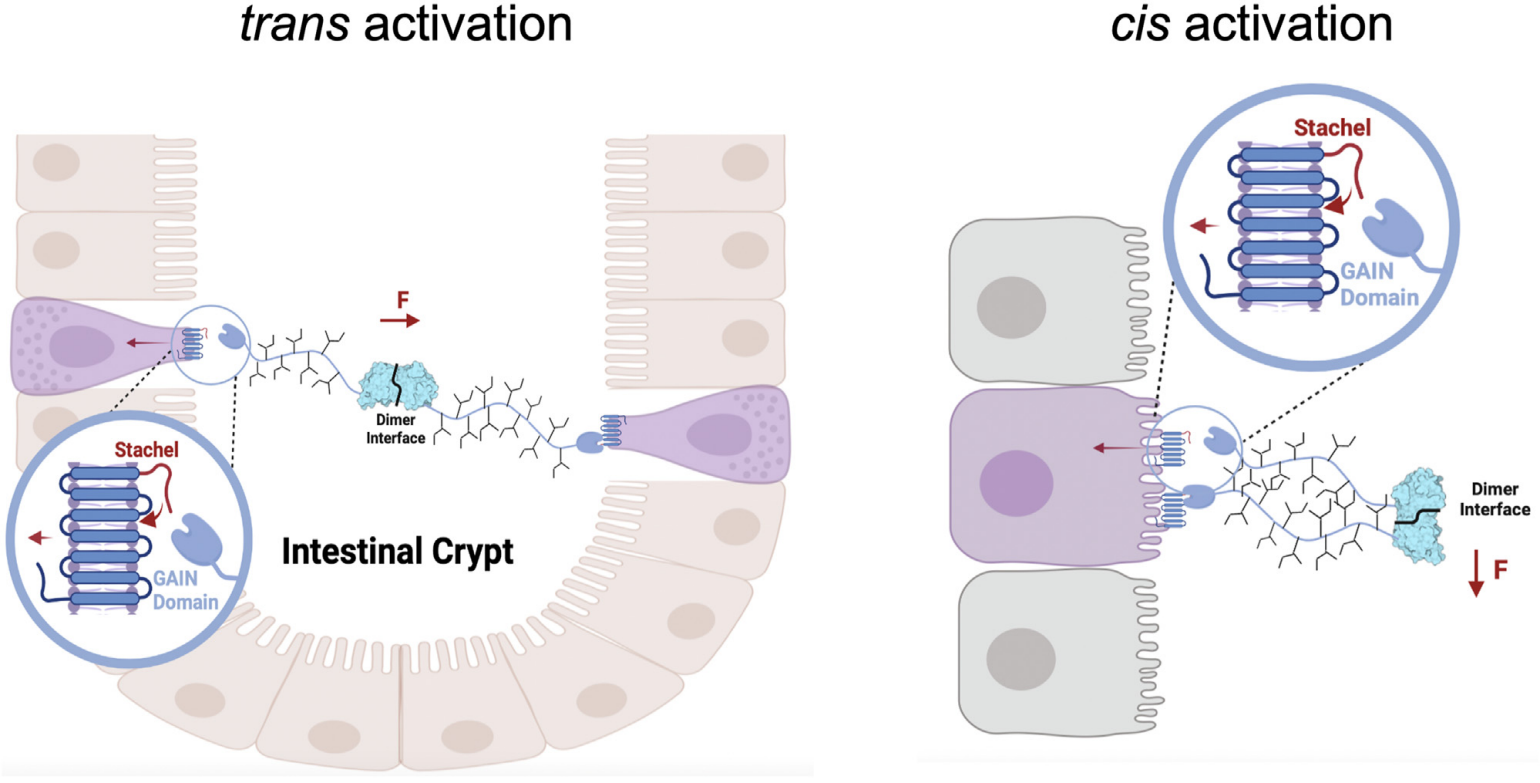
**Scheme of hypothetic homophilic *trans* and *cis* interactions of ADGRG4 in an intestinal crypt.** AGDRG4 may form *trans* dimers *via* homophilic PTX interaction. Upon bowl movements or cell proliferation–mediated changes in the cell-cell distances, forces (F = force) may emerge at the homodimer that are conducted to the GAIN domain (*blue*-colored domain) *via* the stalk. This leads to the exposition of the *Stachel* to activate the 7TM domain. *Black* branches at the stalk indicate its high amount of glycosylation. In a *cis* interaction between ADGRG4 expressed in the same cell, shear forces my lead to receptor activation. Created with BioRender.com. GAIN, GPCR autoproteolysis-inducing domain; PTX, pentraxin.

**Figure 12:**
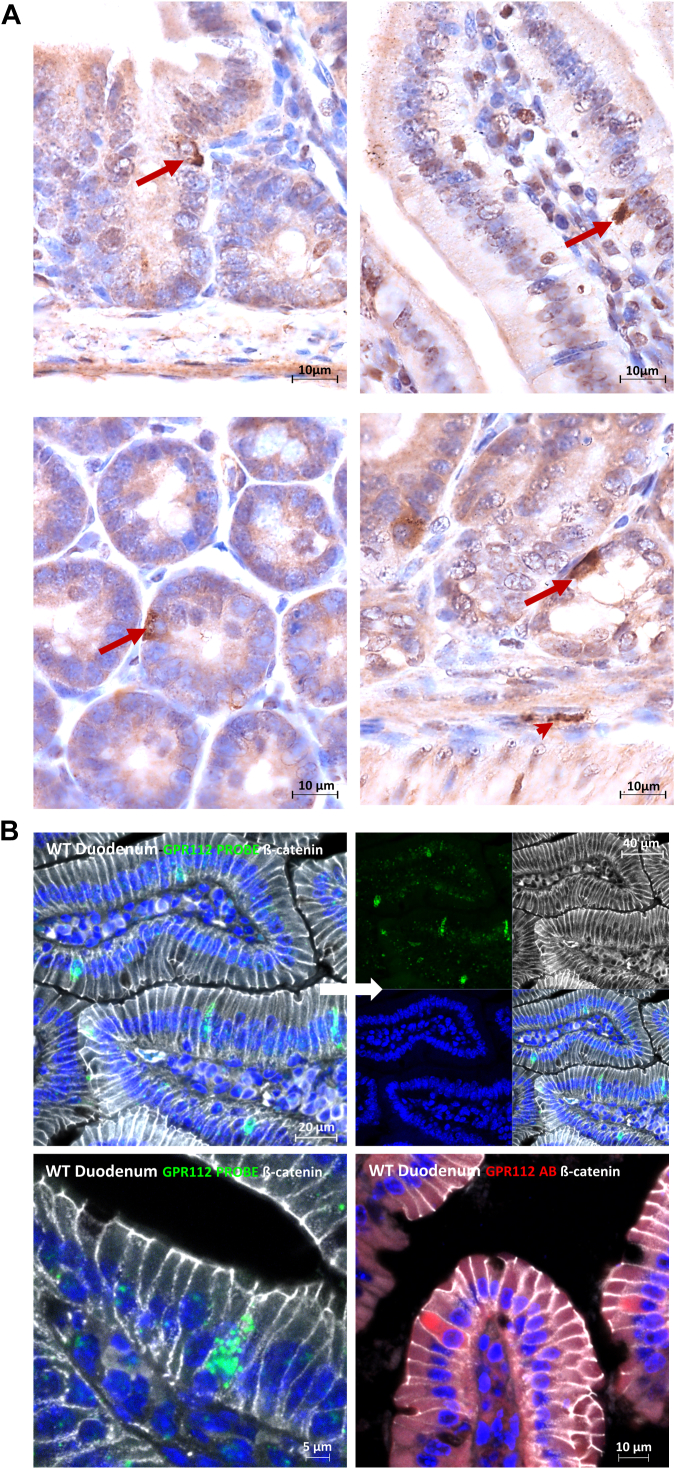
**Detection of ADGRG4 protein and mRNA in small intestinal tissue.***A*, immunohistochemical detection of ADGRG4-positive cells in small intestinal epithelium (*arrows*) and underlying layers (*arrowhead*) using a polyclonal anti-ADGRG4 antibody. *B*, detection of ADGRG4-mRNA (*green*, FISH) or ADGRG4-protein– (*red*, IF) positive cells in small intestinal epithelium highlighted by IF-ß-catenin (*white*) and DAPI-nuclear staining (*blue*). DAPI, 4′,6-diamidino-2-phenylindole; IF, immunofluorescence.

ADGRG4 has been discovered by conventional and single-cell RNA-seq as a specific marker of mouse and human intestinal enteroendocrine/enterochromaffin (EC) cells (31, 61, 62, 63) and Paneth cells (63, 64). EC cells are responsible for serotonin production and secretion and are most abundant in the mucosa of the duodenum (65). The mucosa of the small intestine forms a surface of villi and crypts, which contributes to a large overall surface area gain. The crypts represent deep but very narrow clefts. Here, EC cells are mainly localized (66). EC cells feature a luminal side, which forms microvilli, as well as a basolateral border that is in contact with afferent and efferent nerve terminals located at the lamina propria (67). Similarly, Paneth cells are specialized secretory epithelial cells mainly localized in the crypts of the small intestine. Given the large ECR of ADGRG4, which is potentially able to span a distance of close to 0.5 μm, homophilic *trans* interactions between cells at opposite sides of the crypt lumen may represent a plausible scenario. Together with the general notion of aGPCRs to act as mechanosensors (68, 69, 70, 71), we hypothesize that ADGRG4 acts as a distance sensor within epithelial crypts of the small intestine *via trans* homodimerization (Fig. 11). Movements of the intestinal tract may lead to transient dilations of the crypts. Alternatively, epithelial layer growth may separate *trans*-interacting cells. Once a maximal distance of ∼1 μm is reached (considering two ADGRG4 *trans* dimer molecules), traction forces are generated due to the homodimer interaction. These forces could be transmitted *via* the stalk region to the GAIN domain, resulting in an exposure of the agonistic *Stachel* sequence or activity-relevant isomerization of the tethered agonistic sequence (Fig. 11) (72, 73). To investigate whether such *trans* interaction is feasible, we localized ADGRG4 in small intestine with different methods. First, we applied an anti ADGRG4 antibody directed against the extracellular domain of the receptor. As shown in Figure 12*A*, immunopositive cells were mainly scattered in the intestinal epithelium of crypts and villi. Next, a specific probe to detect ADGRG4 mRNA was used and epithelium cells were costained with an anti-β-catenin antibody (Fig. 12*B*). Again, ADGRG4-expressing cells were mainly scattered in the intestinal epithelium. However, we did not find opposed ADGRG4-expressing cells rejecting the hypothesis of a *trans* homodimerization as the major form of interaction. Therefore, the *cis* homodimerization is more likely. Together with the fact that the highly glycosylated mucin-like stalk between the PTX-like and HRM domain has an increased stiffness and is most likely orientated to the intestine lumen, we propose that ADGRG4 acts as a mechanosensor for shear forces.

In sum, our findings show a specific PTX structure enabling ADGRG4 to homodimerize *via* this domain. Reflecting the unique structure of ADGRG4, the activation mechanism by exposing a tethered sequence in its active conformation (*Stachel*) to the 7TM domain and its highly cell-specific expression, one can assume that this aGPCR is a shear stress mechanosensor in EC and Paneth cells of the small intestine.

## Experimental procedures

### Materials

All chemicals, reagents, and compounds used in this study were purchased from AppliChem, Carl Roth, Sigma-Aldrich, SERVA, and Roche (Basel, Switzerland). Cell culture media and supplement were obtained from Gibco, Life Technologies, Thermo Fisher Scientific. Enzymatic reagents and buffers were purchased from New England Biolabs (Frankfurt a. M., Germany), Thermo Fischer Scientific, and Fermentas. Molecular biology kits were ordered from Qiagen. Prepacked columns for protein purification were purchased from GE Healthcare. Primers were obtained from biomers.net GmbH (Ulm, Germany). To verify cloning results, Sanger sequencing was performed by Eurofins Genomics Germany GmbH.

### Methods

#### Preparation of G4-PTX–like domain

##### Molecular cloning

The complementary DNA encoding for the N terminus of the human ADGRG4 was synthesized by the Thermo Fisher GeneArt service. For subcloning into the pVitro2-EGFP backbone, the following primers were ordered: forward: 5′-TATAACGCGTGCCACCATGAAGGAACACATCAT-3′ (*Mlu*I), reverse: 5′-GTACGTCGACTCATTATTTCTCGAACTGGGGGTG-3' (*Sal*I). The final expression vector comprised the ADGRG4 amino acid sequence 1 to 240 (referred to as G4-PTX) with a GS linker, enteropeptidase site, and StrepII-tag at the C terminus (Fig. S1). The vector backbone also contained a cassette for constitutive EGFP coexpression and a hygromycin resistance as selection marker for stable recombinant mammalian expression.

##### Generation of a stable cell line for large scale G4-PTX expression

A stable HEK293S GnTI^-^ cell line expressing the G4-PTX construct described above was established using hygromycin B resistance. The cell line was cultured in full medium, consisting of Dulbecco’s modified Eagle’s medium supplemented with 10% (v/v) fetal bovine serum, 1% (v/v) GlutaMax, 1% (v/v) non-essential amino acids, and 1% (v/v) penicillin/streptomycin. For large scale expression, aliquots of the cryo-stored cell line were seeded of a single T-75 tissue culture flask and further expanded in T-175 (25 × 10^6^ cells). After reaching confluency, cells were transferred into roller bottles (2125 cm^2^ surface area). The cell line was kept in roller bottles for 2 weeks, with two medium exchanges using reduced medium, which contained only 2% (v/v) of fetal bovine serum. The conditioned media of all passaging steps were stored at 4 °C and pooled. One microliter of BioLock biotin blocking solution (IBA Lifesciences) were added to 1.5 L conditioned medium. The medium was gently stirred for 45 to 60 min at 4 °C and subsequently submitted to centrifugation at 16,000*g* for 30 min. After that, the supernatant was filtered through a 0.22 μm polyether sulfone membrane (Techno Plastic Products). Prior to affinity chromatography, the filtered conditioned medium was tenfold concentrated by an ultrafiltration unit (10 kDa molecular weight cut-off (MWCO), GE Healthcare).

##### Purification

All chromatography steps were performed at 4 °C using the ÄKTAxpress and ÄKTA pure protein purification systems (GE Healthcare). For affinity purification of the ADGRG4 construct, three 5 ml StrepTrap HP (GE Healthcare) were connected to increase the amount of isolated protein per flow through. The washing buffer contained 100 mM Tris pH 8.0, 150 mM NaCl. The elution buffer additionally contained 2.5 mM D-desthiobiotin. After affinity chromatography, the preparation was further polished *via* size-exclusion chromatography. Here, a HiLoad 16/60 Superdex pg 200 (GE Healthcare) was applied for preparative runs. The eluate of the affinity purification was concentrated to a volume between 1 to 2 ml before injection using centrifugal filter units (Amicon, Merck millipore) with a MWCO of 5000 Da. The size-exclusion chromatography buffer contained 20 mM Tris pH 8.0, 150 mM NaCl.

##### Gel-based analyses

SDS-PAGE analysis was conducted after Laemmli. To stain protein glycosylations on SDS-PAGE gels, the periodic acid-Schiff base method was employed (74, 75). Western blot analysis was performed *via* semidry electroblot onto a polyvinylidene fluoride membrane. After blocking the membrane with 3% (w/v) albumin in PBS-T over night, it was treated for 1.5 h with a StrepTactin horse radish peroxidase conjugate (IBA Lifesciences), diluted 1:75,000 in PBS-T. The membrane was washed twice in PBS-T and three times in PBS and readout was enabled using the ECL Select Western Blotting Detection Reagent (GE Healthcare).

#### DLS and SLS

Light scattering measurements were performed using a DynaPro NanoStar device (Wyatt Technologies). The sample was centrifuged prior to measurements for 10 min at 21,000*g*. Three microliters of sample were pipetted into a quartz cuvette. After inserting the cuvette into the device, the system was allowed to equilibrate at 20 °C for 4 min. The integration time for autocorrelation was set to 5 s. Using the software DYNAMICS (https://www.wyatt.com/products/software/dynamics.html, Wyatt Technologies), the autocorrelation function was analyzed by fitting it both in a cumulant and regularized manner. For SLS, a matching buffer blank was measured additionally.

#### X-ray crystallography

##### Crystallization

Crystals suitable for X-ray crystallography were obtained by hanging drop vapor diffusion by mixing 3 μl of protein solution at 3.1 mg/ml with 1 μl of reservoir solution containing 0.1 M Tris pH 8.5, 30% PEG 4000, and 0.2 M MgCl_2_. Crystals between 100 to 200 μm length grew within a week.

##### Data collection and structure determination

Prior to X-ray diffraction experiments, suitable single crystals (min. 50–100 μm length) were transferred for a few seconds to a solution containing the respective crystallization reservoir composition and additional 20% (v/v) glycerol as cryoprotectant. Then, the crystals were snap-frozen in liquid nitrogen on nylon loops connected to goniometer-compatible magnetic mounts and stored in liquid nitrogen–immersed vials until measurement. Crystallographic measurements were performed using synchrotron radiation at beamline P14, operated by EMBL Hamburg at the PETRA III storage ring (DESY). Data were collected at λ = 2.0664 Å (for phasing *via* anomalous dispersion, data not shown) or 0.9762 Å (Table 2) by an EIGER 16M detector. During data collection, the crystal was constantly cooled to 100 K *via* a nitrogen jet. Data reduction was performed by XDS (76). Phasing was achieved by combining molecular replacement and sulfur single-wavelength anomalous diffraction using PHASER and PHASER-EP (77). The initial models were iteratively improved by refinement using PHENIX.REFINE (78) and manual rebuilding in COOT (79). Molecular figures were generated with PyMOL (www.pymol.org).

**Table 2:** Crystallographic statistics of data collection and model refinement

Data collection	
Wavelength/Å	0.9762
Resolution range/Å	51.71—1.36 (1.38—1.36)
Space group	C2
a, b, c/Å	113.12, 40.24, 54.91
β/°	113.9
Total reflections	302,484 (1468)
Unique reflections	43,935 (282)
Redundancy	6.9 (4.5)
Completeness/%	89.7 (19.6)
Mean I/σ(I)	29.6 (2.9)
R_meas_/%	2.8 (52.8)
R_pim_/%	1.1 (22.5)
CC1/2/%	100 (91.7)
Wilson B-factor/Å^2^	19.0
Refinement statistics	
Rwork/Rfree (%)	15.4 (30.1)/18.9 (42.3)
r.m.s.d. bonds (Å)/angles (°)	0.010/1.046
Clashscore	4.37
Ramachandran favored regions/%	98.41
Ramachandran outliers/%	0.00
Rotamer outliers/%	0.56
Number of nonhydrogen atoms	1721
Protein	1613
Heterogen	1
Solvent	107

#### Small-angle X-ray scattering

SAXS measurements were performed using synchrotron radiation at beamline P12, operated by EMBL Hamburg at the PETRA III storage ring (DESY) (80). Data were collected at 1.23987 Å using a Pilatus 6M detector at 3.0 m sample-detector distance. The sample cell consisted of a horizontal thermostated (278–323 K) quartz capillary with 50 μm thick walls and a path length of 1.7 mm. The samples (30 μl) continuously flowed through the quartz capillary to reduce X-ray radiation damage. To minimize aggregation due to radiation damage, 3 to 5% (v/v) glycerol was added to the samples.

The SAXS data were automatically processed on-site by the SASFLOW suite (81). Processing involves radial averaging of the two-dimensional scattering pattern, normalization against the transmitted beam intensity, detection and exclusion of frames suffering from radiation damage, and buffer background subtraction.

##### Modeling of all-atom ensembles and SAXS data analyses

For SAXS data analysis, amino acids and glycans that were missing in the crystal structures were added *in silico*. First, C_α_-traces of the unstructured loop as well as the C-terminal portion were modeled using RANCH (44) from the ATSAS suite (version 3.1.3) (82). The C_α_-traces were converted to full-atom models using MODELLER (version 10.4) (83). The atom coordinates obtained from the crystal structure and the C_α_-trace were fixed for side chain modeling, except for connecting residues, which were allowed to move to avoid nonsensible conformations.

Next, high-mannose–type glycans were attached to residues N166 and N233 using ALLOSMOD (84). Three pools of models with different glycosylation patterns were generated: (i) without glycosylation, (ii) glycosylated at the more likely N-glycosylation site N233, and (iii) glycosylated at N166 and N233 (39). For each model pool, dimeric G4-PTX were generated by superimposing the monomer models on the dimeric arrangement. Finally, models with severe clashes were filtered out.

The final pools consisted of (i) nonglycosylated G4-PTX with 5855/2812 monomer/dimer models, (ii) G4-PTX glycosylated at N233 with 5273/2529 models, and (iii) G4-PTX glycosylated at N166/N233 with 4240/1784 models. To determine the molar fractions of monomer and dimer that best fit the experimental data, either EOM (NNLSJOE) or OLIGOMER from the ATSAS suite were used. NNLSJOE (44) is an ensemble optimization program that selects an ensemble from the pool to represent the experimental SAXS data curve. The scattering contribution of each model of the ensemble is represented by a volume fraction factor, from which the molar fraction of each component is deduced. The sum of the molar fractions of all monomers (or dimers) in an ensemble yields the mixtures’ overall molar monomer (dimer) fraction. The required form factors of each model from all three pools were calculated using FFMAKER. For analyses with OLIGOMER, theoretical scattering curves of all models in the three pools were calculated with CRYSOL (85). Averaged curves for either monomeric or dimeric G4-PTX were obtained with programs from the ATSAS suite. OLIGOMER (45) was then used to derive the molar fractions of both species.

##### Homodimer affinity determination

The K_D_ of homodimer formation was obtained by nonlinear regression of both the experimentally obtained R_g_-values of the G4-PTX concentration series and the molar dimer fractions determined by ensemble optimization modeling.

For a homodimer equilibrium, the following nonlinear relationship exists between the apparent R_g_ and the molar fraction of monomer f_M_ (86, 87):

(1) $$R_{g}^{2}\;=\;\frac{f_{M}R_{g,M}^{2}\;+\;2\left(1\;-\;f_{M}\right)R_{g,D}^{2}}{2\;-\;f_{M}}$$

with

(2) $$f_{M}=\frac{\left[M\right]}{{\left[M\right]}_{0}}$$

R_g,M_ and R_g,D_ represent the theoretical R_g_-values of the monomer and dimer structure, respectively. These theoretical R_g_ were extracted as averages of the model pools created for ensemble optimization. The following R_g_-values for monomer and dimer were employed, assuming different degrees of N-glycosylation as described for the model pool creation above:

(i) not glycosylated: R_g,M_ = 21.94 Å, R_g,D_ = 29.55 Å; (ii) glycosylated at N233: R_g,M_ = 22.50 Å, R_g,D_ = 30.38 Å; (iii) glycosylated at N166/N233: R_g,M_= 23.05 Å, R_g,D_= 30.84 Å.

The molar monomer concentration [M] in a homodimer equilibrium depends on the dissociation constant K_D_ and the total molar protein concentration [M]_0_ by the law of mass action:

(3) $$\left[M\right]=\frac{1}{4}*\left(-K_{D}+\sqrt{K_{D}^{2}+8{\left[M\right]}_{0}K_{D}}\right)\text{.}$$

From Equations (1), (2), (3), a relation between R_g_^2^ and [M]_0_ was derived, which was employed as a fit function for nonlinear regression:

(4) $$R_{g}^{2}=\frac{R_{g,M}^{2}\left(A-K_{D}\right)+R_{g,D}^{2}\left(8{\left[M\right]}_{0}+2K_{D}-2A\right)}{8{\left[M\right]}_{0}+K_{D}-A}$$

with A representing the term:

(4a) $$A=\sqrt{K_{D}^{2}+8{\left[M\right]}_{0}K_{D.}}$$

For nonlinear regression of the dimer mole fractions determined by ensemble optimization modeling, the mol fraction of dimer was calculated as:

(5) $$x_{D}=\frac{n_{D}}{n_{M}+n_{D}}=\frac{\left[D\right]}{\left[M\right]+\left[D\right]}=\frac{1}{1+\frac{K_{D}}{\left[M\right]}}$$

Inserting (3) yields:

(6) $$x_{D}=\frac{x_{D,\max}}{1+\frac{4K_{D}}{A-K_{D}}}$$

with x_D,max_ = 1. For fitting the concentration dependence of the ensembles obtained from EOM analysis, x_D,max_ was fitted as variable, as the dimer mole fraction did not approach to 1 at high protein concentrations (Fig. S7).

#### Phylogenetic analysis

##### Cluster analysis

The sequences of PTX-like domains were taken from NCBI and aligned using MUSCLE implemented in MEGA11 (88, 89). The evolutionary history was inferred by using the Maximum Likelihood method and JTT matrix-based model (90). The bootstrap consensus tree inferred from 1000 replicates (91) is taken to represent the evolutionary history of the taxa analyzed. Branches corresponding to partitions reproduced in less than 50% bootstrap replicates are collapsed. The percentage of replicate trees in which the associated taxa clustered together in the bootstrap test 1000 replicates are shown next to the branches (ibid.). Initial tree(s) for the heuristic search were obtained automatically by applying Neighbor-Join and BioNJ algorithms to a matrix of pairwise distances estimated using the JTT model and then selecting the topology with superior log likelihood value. This analysis involved 102 amino acid sequences. There were a total of 350 positions in the final dataset. Phylogenetic analyses were conducted in MEGA11 (88, 89).

##### Shannon-entropy conservation scores

An MSA of 59 mammalian ADGRG4 orthologs was generated using MUSCLE. Based on this MSA, the Shannon entropy of each aa residue was determined as a measure of evolutionary conservation (92). Subsequently, these Shannon entropy scores were plotted onto the crystal structure, employing the Protein Variability Server (93).

#### Structure prediction

The ColabFold (58) implementation of AlphaFold algorithms (46) was used to predict dimer structures. Calculations were run on the ColabFold notebook AlphaFold2.ipynb. MSAs were generated *via* MMseqs2 (58) using sequences from UniRef. AlphaFold2-multimer was used for structure prediction and the "paired+unpaired" mode (ibid.) was chosen for complex prediction.

#### Cell localization experiments of ADGRG4 (GPR112) in murine duodenum

##### Immunohistochemical and immunofluorescence staining

Small intestine was harvested from mice and fixed overnight at 4 °C in 4% phosphate-buffered formalin. After thorough rinsing with tap water, the tissue was dehydrated in a series of graded alcohols, passed through xylene, and embedded in paraffin wax. The embedded tissue was cut into 10 μm thick sections, deparaffinized in xylene, and rehydrated in a series of graded alcohols. Prior to IHC-anti-GPR112-staining, hydrated sections were heated in sodium citrate buffer (pH 6.0, 10 min) to retrieve antigens, immersed in 3% H_2_O_2_ in PBS (pH7.4, 10 min, room temperature [RT]) to quench endogenous peroxidase activity, and incubated with 5% normal goat serum (30 min, RT) to reduce background staining due to nonspecific interactions between the secondary biotinylated goat anti-rabbit antibody and the section surface. Primary anti-GPR112 polyclonal antibody (cpa3095, Cohesion Biosciences) was diluted 1:100 in antibody buffer (PBS, pH 7.4 containing 0.5% bovine serum albumin and 0.3% Triton X-100) and applied overnight at 4 °C in a humidified chamber. The anti-GPR112 polyclonal antibody was generated by immunization of rabbits with a keyhole limpet hemocyanin-conjugated synthetic peptide encompassing a sequence within the center region of human GPR112. The company provides verification *via* Western blot on human, mouse, and rat ADGRG4/GPR112 orthologs. Immune complexes were visualized by successive incubations with biotin-conjugated goat anti-rabbit secondary antibody (1 h, RT), avidin-biotin-peroxidase complex (Vectastain, 30 min, RT), and a mixture of 3,3′-diaminobenzidine and urea-hydrogen peroxide (SigmaFast DAB tablets, Sigma-Aldrich, 30–60 s, RT). The peroxidase/DAB reaction was stopped by rinsing the sections with 0.05 M Tris–HCl buffer, pH 7.4. Finally, immunohistochemically labeled sections were counterstained with Mayer's hemalum, dehydrated, and embedded with Roti-Histokit (Carl Roth). For immunofluorescence labeling, a 1:100 dilution of primary polyclonal anti-GPR112 antibody was applied together with primary monoclonal anti-ß-catenin antibody (1:100, #610154, BD Biosciences) on antigen-retrieved and background reduced sections overnight at 4 °C. Immune complexes were detected with a mixture of species-matched Alexa-568– and 647-conjugated secondary antibodies (1:400, Invitrogen), whereas nuclei were counterstained with DAPI (4′,6-diamidino-2-phenylindole, Serva). Finally, sections were embedded in fluorescent embedding medium from Dako (Aligent). Fluorescence images were captured using an LSM700 confocal laser scanning microscope (Carl Zeiss AG).

##### Fluorescence *in situ* hybridization

The RNAscope Multiplex Fluorescent Reagent Kit v2 (Advanced Cell Diagnostics [ACD]) was used to localize ADGRG4 mRNA on small intestinal sections. Tissue was fixed with 4% phosphate-buffered formalin for 24 h and embedded in paraffin wax. Sections of 10 μm were made from the paraffin-embedded tissue samples and mounted on Superfrost slides. ADGRG4 RNA labeling was performed using a commercially purchased probe from ACD according to the manufacturer's instructions. The 1:50 diluted probe was incubated on the tissue sections for 2 h at 40 °C in a manual HybEZ II assay hybridization system (ACD). Probe remaining on the tissue sections was amplified and labeled with a single Opal 520 fluorophore at a dilution of 1:750 (OP-001001, Akoya Biosciences). After RNA labeling, combined ß-catenin Alexa 647-immunofluorescence (IF) staining and DAPI nuclear counterstaining was performed as described above. The now fluorescence *in situ* hybridization-IF–stained sections were embedded and analyzed under the LSM 700 confocal laser scanning microscope (see above).

## Data availability

The crystal structure of the ADGRG4 PTX-like domain is publicly available from the Protein Data Bank with accession code 8B55 (www.rcsb.org).

Small-angle X-ray scattering data on ADGRG4 PTX-like domain, including the respective ensemble models, are publicly available from the Small Angle Scattering Biological Data Bank (www.sasbdb.org). The accession codes for each dataset are stated in Table 1.

All other data relevant to this study are provided in the main text and the supplementary information.

## Supporting information

This article contains supporting information (46, 58, 94, 95, 96, 97).

## Conflict of interest

The authors declare that they have no conflicts of interest with the contents of this article.
